# One Health Research in Northern Tanzania – Challenges and Progress

**DOI:** 10.24248/EAHRJ-D-16-00379

**Published:** 2017-03-01

**Authors:** Georgia Ladbury, Kathryn J Allan, Sarah Cleaveland, Alicia Davis, William A de Glanville, Taya L Forde, Jo E B Halliday, Daniel T Haydon, Gibson Kibiki, Ireen Kiwelu, Tiziana Lembo, Venance Maro, Blandina T Mmbaga, Theonest Ndyetabura, Jo Sharp, Kate Thomas, Ruth N Zadoks

**Affiliations:** a Institute of Biodiversity Animal Health and Comparative Medicine, College of Medical Veterinary and Life Sciences, University of Glasgow, Glasgow, UK; b School of Geographical and Earth Sciences, College of Science and Engineering, University of Glasgow, Glasgow, UK; c Kilimanjaro Clinical Research Institute, Good Samaritan Foundation, Moshi, Tanzania; d East African Health Research Commission, Arusha, Tanzania; e Kilimanjaro Christian Medical Centre, Good Samaritan Foundation, Moshi, Tanzania; f Centre for International Health, University of Otago, Dunedin, New Zealand

## Abstract

East Africa has one of the world's fastest growing human populations—many of whom are dependent on livestock—as well as some of the world's largest wildlife populations. Humans, livestock, and wildlife often interact closely, intimately linking human, animal, and environmental health. The concept of One Health captures this interconnectedness, including the social structures and beliefs driving interactions between species and their environments. East African policymakers and researchers are recognising and encouraging One Health research, with both groups increasingly playing a leading role in this subject area. One Health research requires interaction between scientists from different disciplines, such as the biological and social sciences and human and veterinary medicine. Different disciplines draw on norms, methodologies, and terminologies that have evolved within their respective institutions and that may be distinct from or in conflict with one another. These differences impact interdisciplinary research, both around theoretical and methodological approaches and during project operationalisation. We present experiential knowledge gained from numerous ongoing projects in northern Tanzania, including those dealing with bacterial zoonoses associated with febrile illness, foodborne disease, and anthrax. We use the examples to illustrate differences between and within social and biological sciences and between industrialised and traditional societies, for example, with regard to consenting procedures or the ethical treatment of animals. We describe challenges encountered in ethical approval processes, consenting procedures, and field and laboratory logistics and offer suggestions for improvement. While considerable investment of time in sensitisation, communication, and collaboration is needed to overcome interdisciplinary challenges inherent in One Health research, this can yield great rewards in paving the way for successful implementation of One Health projects. Furthermore, continued investment in African institutions and scientists will strengthen the role of East Africa as a world leader in One Health research.

## INTRODUCTION

Eastern Africa has one of the highest predicted human population growth rates on the planet.^1^ It is also home to some of the world's largest wildlife populations, many of which live in close proximity to people and livestock—directly linking people's lives to the health of livestock and the natural environment. The interdependence of human, animal, and environmental health—termed One Health^2^—creates both unique challenges and opportunities that could make East Africa a world leader in the development of One Health research and policy. The instinctive understanding of these interdependencies, together with the maturing of health research institutions and the establishment of One Health research consortia in Africa, provides an opportunity for African scientists and institutions to lead the global One Health agenda. Moreover, in East Africa, One Health is also embraced by policymakers in some countries, such as Tanzania and Kenya, to the extent that they have established One Health departments within their governments.^3,4^ These intersectoral units set out not only to establish the interdisciplinary collaborations required for effective responses to emerging zoonotic diseases, but also to recognise the challenges of endemic zoonoses that continue to threaten human and animal health.^5^ The growing awareness of endemic zoo-noses as an important cause of common human disease syndromes, including fever,^6–10^ emphasises the relevance of One Health as a daily health concern, beyond outbreaks of “newsworthy” diseases such as Ebola, SARS, or highly pathogenic avian influenza (HPAI).

One Health research requires the collaboration of multiple disciplines, including medical, veterinary, and biological scientists alongside quantitative and qualitative social scientists. As dramatic advances were made in medical science and the practice of human medicine in industrialised societies in the 20th century, medicine, veterinary medicine, and social sciences developed as largely separate entities, with separate professional and scientific organisations. The growth of One Health publications has surpassed that of life science publications overall, suggesting an increased uptake of interdisciplinary approaches.^11^ Even so, publication silos and differences in attitudes and best practices between and within biological and social sciences continue to exist. These differences impact interdisciplinary research, not just in terms of theoretical and methodological approaches, but also with regard to the operationalisation of the projects through, for example, ethical approvals, consent procedures, laboratory requirements, and authorship of publications.

In this contribution, we share our experiential knowledge gained from implementation and management of One Health research programs in northern Tanzania, concentrating on bacterial zoonoses research—conducted by the University of Glasgow, in collaboration with a wide range of Tanzanian and international partners—and on the development of bespoke zoonoses laboratory space at the Kilimanjaro Clinical Research Institute (KCRI) in Moshi. We begin with a discussion of ethical approval, which needs to be in place before field or laboratory work commences, and present some of the issues that may arise when working across social and biological disciplines. We continue with a description of some of the challenges in developing field-based and laboratory capacity for One Health research, where differences between human and animal health processes and policies come to the fore. Finally, we use case examples from current research projects to demonstrate research opportunities when such challenges are overcome and One Health research can be effectively implemented.

## ONE HEALTH RESEARCH ETHICS

Regardless what type of human or animal research is conducted, the safety and protection of research participants should be paramount, and ethical committees and review procedures have a key role in safeguarding them.^12,13^ Owing to its interdisciplinary, interinstitutional, and, frequently, international nature, ethical review of One Health projects can pose special challenges, an issue that is also recognised in relation to review of funding applications for One Health research.^14^ Different disciplines draw on norms, methodologies, and terminologies that have evolved and become accepted within their respective institutions. As ethics committees are usually embedded within each of these institutions, the review process for interdisciplinary research can be problematic. An ethics committee whose members are familiar with biomedical research may struggle to assess research that is based on social science and vice versa. For example, in the initial review of one of our projects, focused on understanding the dairy value chain in northern Tanzania, members of a biomedical ethics committee were unfamiliar with the use of standard social science research terminology—such as “actor” and “purposive sampling”—which caused an obstacle to approval. The reviewers also expressed a strong preference for written over verbal consent, as is standard in medical research. While written consent is also common practice across the social sciences, it is not always appropriate—such as within communities with high illiteracy rates. When written consent cannot be given, the discipline norm is to record verbal consent. Rigid ethical regulations that insist on a written method of consent could preclude data collection in situations where such procedures would be challenging to apply.^15^ A consequence of this could be that research on marginalised groups becomes more difficult or even impossible to carry out, which could lead to unethical outcomes of an ethical review process.^15^ Another layer of complexity is added by the fact that many One Health projects involve multiple institutions, often in multiple countries, and often with their own or even multiple consenting procedures, for example human subjects vs. animal research. For some of our bacterial zoonoses projects, ethical review was required by up to 7 committees across 4 countries. Questions about ethical jurisdiction can arise when multiple international and local institutional ethics committees involved in ethical review take different positions.^16^

### Human Subjects Research

Informed consent is the cornerstone of ethical scientific research involving people, whether as research subjects or as owners of animal research subjects. The informed-consent process is comprised of 3 key tenets^17^: firstly, the participant must be given full and transparent information about the research and their rights in an easily understandable manner; secondly, the participant must comprehend what is being asked of him or her; and lastly, the participant must freely agree to take part in the research.

The convention for the first tenet—provision of information—is to produce written documents, such as information sheets and consent forms, for participants. In our research, these typically cover the study objectives, potential risks and benefits of participating, research organisations and funding bodies, confidentiality, who has reviewed the study, what will happen to the results, and the researchers’ contact details. The forms also include statements encouraging participants to ask questions and informing them that they can withdraw from the research at any time. In Tanzania, the adult literacy level is 68%,^18^ indicating that reliance on written documentation to convey information would likely exclude a considerable proportion of potential study participants. This problem is made worse by the fact that One Health research frequently involves groups—such as women, rural populations, smallholder farmers, or informal traders—who may have literacy rates below the national average. Presenting written information to a person with a low level of literacy may cause embarrassment, something an ethical researcher should strive to prevent rather than to precipitate. In our experience, even literate participants often felt intimidated rather than reassured by our consent documents. For example, our Tanzanian research assistants reported the high level of detail on our information sheets—especially the details of multiple research partners with formal institutional logos—sometimes caused concern about hidden goals of the research. Potential participants refused to believe that so much paperwork could be associated with something as straightforward as taking part in an interview or survey. On multiple occasions, people mistook us for members of a group rumoured to have malicious intent, a problem other researchers also encountered.^19^ Additionally, some of the information provided on the forms—such as contact information for UK-based researchers—was not meaningful to participants and thus risked “information overload” for literate and illiterate participants alike. This often resulted in important messages, such as the right to refuse or withdraw participation, being buried in other information. Bhutta drew attention to the drawbacks of written information documents, arguing that they served largely to satisfy researchers’ concerns over the legality of the informed consent process rather than the needs of the study participants.^17^ Requesting written confirmation of participants’ consent, such as a signature, may also pose difficulties when working with individuals or groups with low literacy. In theory, this issue could be solved by taking participants’ thumb prints, but in a study of U.S.- and developing country-based researchers, respondents considered oral consent preferable to written consent, both for sharing information and for documenting consent.^20^ Likewise, for a survey of informal milk vendors in Kenya, participants chose to give consent verbally (140 of 230, 61%) rather than by signature (39%), and none provided thumbprints.^21^

Considering the second tenet—comprehension—we found it difficult to assess the degree to which consent was genuinely informed even after the agreed informed-consent procedure had been dutifully followed. The consent process tended to take place quickly because the research was taking people away from other activities and because participants often became visibly bored when full details were read out slowly. Moreover, although we attempted to clearly explain the research activities, the gap between the researchers’ lived experiences and those of the participants was vast and created communication challenges. Despite high rates of consent, we were not always confident that participants had genuinely understood the research goals, nor had they had time to reflect on whether or not to take part in the study. This is not a new problem in development research. The standard consent process of a single meeting between investigator and volunteer may be insufficient for adequate comprehension of informed consent.^22^ Indeed, Sreenivasan argues that full comprehension is rarely achieved in research and should be considered an ethical aspiration rather than a minimum standard,^23^ claiming that if full comprehension was a necessary condition of valid consent then much scientific endeavour would be impossible. To protect prospective research participants, their level of understanding can be assessed prior to consenting. Cooper and colleagues used comprehension and engagement scores to test the effectiveness of 3 communication tools—written documents, illustrative photographs, and illustrative cartoons—when providing study information to 22 Tanzanian livestock keepers prior to consent.^21^ Cartoons were associated with significantly higher engagement scores, highlighting the usefulness of alternative means of information provision.

**Photo 1.**
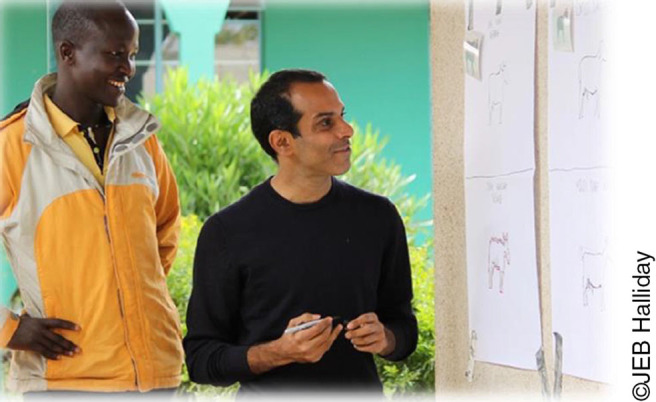
Development of bespoke cartoons in consultation with Tanzanian end-users to create culturally appropriate communication tools for One Health research.

Finally, the third tenet—freely agreeing to consent—posed several problems. It proved difficult in some circumstances to identify who should be providing consent. Western ethical models place emphasis on the individual; however, in many African and other low- and middle-income country (LMIC) contexts, additional consent at the family or community level may be necessary.^20,23–25^ In northern Tanzania, we sought permission of ward officials or community leaders, such as village chairs, prior to conducting key informant interviews. This was not required by our ethics committees but was recommended as standard practice by researchers with experience working in the area. However, even this level of contingency was not always sufficient. For one interview we visited a 19-year old informal milk trader at his family's home. We were accompanied by the village chair who had arranged the interview with the trader's prior agreement. Halfway through the interview we were interrupted by the trader's angry father, irate that we were holding an interview on his compound without his express consent. The village chair's presence made no difference and we had clearly violated a social or familial hierarchical norm. Such situations have the potential to compromise the safety and wellbeing of both researchers and participants, thus breaching a fundamental bioethics principle to do no harm.

A further difficulty with the third tenet is ensuring that consent is truly voluntary. Power relations can have coercive effects on consent. The presence of an outsider, the endorsement by a community leader, or the gender of a participant could all serve to socially or politically pressure participants who feel they ought to participate. The social desire to please others—whether conscious or subconscious—is particularly strong if those others are perceived as having power or control.^26^ In some instances, women refused to be interviewed if their husbands were not home to consent, although in some cases this may have been a strategy for tacit refusal.^27^ Financial incentives can also be coercive. We attempted to avoid this by not offering any remuneration other than the reimbursement of travel expenses. Although we made an exception when reimbursing local officials for the time and costs of arranging research activities, as is the local norm for recruiting these community gatekeepers, this practice could have been construed as reinforcing power imbalances that discriminate against the poor by unintentionally implying a poorer person's time has less value than the time of those privileged with education, wealth, and social status.

### Animal Research

Use of animals in research comes with its own ethical challenges, especially where multiple international partners are involved. Animal research performed by researchers at the University of Glasgow is governed by the Animals (Scientific Procedures) Act 1986, implemented under European Union (EU) Directive 2010/63/EU, which sets out measures for the protection of animals used for scientific purposes.^28^ Research funded by United Kingdom (UK) funders or conducted by UK universities is required to comply with this Act. However, while the UK Home Office provides detailed guidance on implementation of the Act within the UK, it does not take into consideration implementing work overseas.^28^ The EU directive—largely developed with housed laboratory animals in mind—does not necessarily make adequate provision for research on other animals or in other settings.^29^ These limitations make it difficult for researchers and local animal welfare ethical review bodies (AWERB) in the UK to interpret the guidance in international contexts. Few African institutions have AWERBS or institutional animal care and use committees (IACUCS) to review animal research within One Health proposals. Instead, issues relating to animal use in African countries are routinely addressed by AWERBs or IACUCs of partner institutions in Western countries. As a result, the policies and practices largely reflect the values and concerns of societies in those countries,^30^ which may not necessarily be the same as those in African or other non-Western societies. While reviews of animal care and use in LMICs often emphasise the need for standards to meet those evolving in Western countries,^31^ in this commentary we question whether and why decisions made in Western countries are given precedence as they may, in some cases, be inappropriate and ethically unacceptable to non-Western societies in Africa and elsewhere.

Most studies on attitudes toward animal research have been carried out across a narrow range of high-income countries in Europe and North America,^32,33^ while little research has been conducted on the attitudes of people in lower-income and agricultural-based societies, especially in the LMICs. Furthermore, such studies tend to focus on a limited number of animal species including non-human primates; companion animals, such as dogs and cats; and laboratory animals used in biomedical research, such as mice and rats. Little attention has been given to species involved in One Health research such as livestock and wildlife. Contact with animals is likely to foster emotional attachment and empathy towards the species involved and affects attitudes towards animal use in research.^34,35^ In Western societies, most human–animal contact involves companion animals or equids and the emotional attachment formed with those animals is reflected in the higher level of scrutiny given to the use of these species in research.^28^ In those societies people have little direct contact with livestock species, which tends to preclude them from becoming objects of people's positive affections—resulting in attitudes dominated by the utilitarian view of livestock as a source of products.^36^ In contrast, in rural and peri-urban communities in African countries, which comprise a large proportion of the population, people live in close contact with livestock. In Maasai pastoral societies, where we conduct research on bacterial zoonoses, many aspects of spirituality and mythology focus on the importance of cattle, which are seen as manifestations of the god, *Ngai*.^37^ Additionally, livestock and pastoral livelihoods are crucial to Maasai identity as well as economic, social, and cultural capital.^38^ Because the Maasai and other livestock-keeping societies often have complex spiritual and practical relationships with livestock, a strong case can be made for greater understanding of these relationships when making decisions on animal use in research. For example, during recent field trials to evaluate the efficacy of a new vaccine against malignant catarrhal fever (MCF) in Tanzania,^39^ residents of the Maasai communities—where the trials were being conducted—expressed considerable misgivings about exposing cattle to the risk of a potentially fatal disease through contact with wildebeest, even though the cattle in question had been purchased from outside the community specifically for the trial and were unknown to the community. Although the Tanzania study protocol followed UK trial guidelines^40^ and was compliant with the UK Animals (Scientific Procedures) Act 1986 for euthanasia of animals with severe clinical disease, euthanasia of healthy animals that posed no disease risk to people or other animals was not carried out at the end of the trial. Euthanasia was considered socially unacceptable in the Maasai communities and the unnecessary loss of valued animals was likely to have caused considerable concern among community members. Even in Western societies, the harms and benefits of culling of healthy animals are being re-evaluated, particularly in the context of One Health, where not just human health but also animal and environmental health are given consideration.^2^

### Incorporating the Plurality of Ethical Perspectives

One Health research is challenging because it brings many ethical considerations simultaneously to the fore: it is interdisciplinary and often international, endemic zoonoses disproportionately impact poor and vulnerable populations living in LMICs, and zoonoses research inherently covers both animals and humans. The plurality of processes—with different standards and procedures across committees assessing medical, veterinary or social science research at different organisations in multiple countries—often cause considerable delays to projects due to the varied and sometimes contradictory demands of the different ethical bodies. The multiplicity of standards and ethical procedures and practices is neither suitable nor adequate for this complex area of research, and innovative solutions are urgently required. Although there are legal constraints on modifications of ethics processes, we would like to suggest that the balance between disciplines and between legal and ethical aspects of the process may need to be reconsidered. More specifically, our first suggestion is that One Health applications should be reviewed by a single ethical review committee, rather than separate committees for each discipline. Such committees should be interdisciplinary—including experts in human medicine, animal health, and social sciences. Those committees would become familiar with the language, culture, and standards of the different disciplines—and consider all aspects of an interdisciplinary One Health proposal in a single assessment. While there is international agreement on the need for ethical conduct, consent procedures, and oversight in human subjects research, uniform implementation of Western guidelines for ethical conduct may create conflict between the “spirit” and “letter”—the underlying principles and technical implementation—of such guidelines in other countries or cultures.^12,13,41^ Our second suggestion, therefore, is that ethical approvals from the country where the research is to be conducted should be considered as possible basis for ethical approval by the country where the funding originates. In our example, this could mean that the University of Glasgow would consider waiving the need for ethical approval if such approval were already granted by KCRI and the National Institute for Medical Research (NIMR), as the relevant local and national institutions, respectively, in Tanzania. This would prevent “ethical colonialism”, whereby ethical standards from one country are imposed on another country without considering the cultural appropriateness of the ethical requirements. Already, waivers for ethical review may be granted by institutions within a single country—for example, across different universities in New Zealand—and such an approach could be adopted more widely, albeit with consideration of applicable legal constraints. Finally, this system would also require development of appropriately trained interdisciplinary ethics committees in LMICs. The development of an ethical and compliance infrastructure should be included in capacity-building initiatives, which, more often than not, focus on physical spaces and equipment or on technical and scientific skills, rather than on institutional, administrative, or ethical-research infrastructure.

## ONE HEALTH RESEARCH LOGISTICS

A major aim of zoonoses research is to generate data that informs understanding of the linkages between human and animal infection. To obtain insights into transmission processes between species and populations, epidemiological studies need to capture data on human and animal populations and their connectivity. This requires the design and delivery of multiple linked epidemiological studies and careful consideration of the aims and intended outputs from each component part. Because of the differences in the structure and distribution of species and populations, the sampling units or strategies appropriate for one species or population may not apply to others, especially when contemporaneous collection of samples or data from multiple species is needed. For example, while hospital- or school-based approaches may be the most efficient and cost-effective way to obtain samples from human populations, comparable sampling strategies for animal species are more difficult to find. Where they do exist, such as through veterinary surveillance or sampling at livestock markets or slaughterhouses, human behavioural factors linked to disease reporting or to the sale of particular livestock may make them poorly representative of the true disease situation in animals.^42,43^ Household-based sampling strategies where data are gathered from individuals of multiple species present at the same place and time provide a way to gather fine-scale data from linked populations, but these efforts are resource and time intensive, and defining a household may be a challenge in its own right.^44^ Careful evaluation of the nature of the linkages between populations, the scales at which they are observed and sampled, and the trade-offs between optimal study design and feasibility is crucial. The increasing availability of online databases with molecular typing or sequencing data for pathogens may provide a false sense of comparability across studies, as the standardised format of such data masks underlying differences in epidemiological origins and reporting zeal.^45^ This may contribute to erroneous interpretations regarding host-adaptation of pathogens or transmission potential between host species,^46^ thus emphasising the need for epidemiologically appropriate study designs in One Health research.

Gaining access to appropriate laboratory space for processing of samples from people and animals can be difficult due to differences in regulation, location, and operation of human and animal diagnostic laboratories. For example, quality assurance/quality control check lists, such as those used by the U.S. National Institutes of Health Division of AIDS, may include questions on the presence of animal samples in the laboratory, with the implication that animal samples pose an inherent biohazard that could affect human infectious disease research. This is at odds with the fact that the level of containment needs to reflect the hazard level of the pathogen rather than the host species. For example, *Bacillus anthracis* is a Hazard Group 3 organism regardless of its origin. For some infections, such as HPAI, where false positive results could trigger a pandemic influenza alert, international guidelines explicitly state that human and animal samples should not be processed in the same laboratory.^47^ Some would argue that the hazard of cross-reactivity with unrecognised diagnostic targets in samples of animal origin could lead to false-positive results and that prudence dictates separation of laboratories at all times. A counter argument would be that such cross-reactivity can also occur within human samples, meaning that separation of laboratories does not fully resolve this issue.^48^ Cross-contamination or cross-reactivity, while highly undesirable, would be less impactful for endemic than epidemic infections. Implementation of good working practices supported by standard operating procedures, risk assessments, and staff-induction protocols should be put in place to minimise cross-contamination risks in microbiological and molecular laboratories, irrespective of the origin of samples. In our experience, sensitisation and training about zoonotic disease research was key to overcoming initial resistance, building consensus, and gaining acceptance of work on projects involving human and animal samples for laboratory accreditation bodies and staff alike.

An unintended consequence of the separation of laboratories and staff processing human and animal samples is lack of communication. For example, our research team was involved in anthrax diagnostics on animal-derived material and was not aware of the availability of molecular diagnostics for anthrax in human samples in an adjacent laboratory until a discussion about diagnostics was sparked by the occurrence of human anthrax in northern Tanzania.^49^ Such issues could be resolved by regular communication between staff, staff training and cross-training, and the collocation of human and veterinary diagnostic facilities and experts. To allow for the processing of animal-derived material from our One Health projects in northern Tanzania, a physically separate containment level-2 laboratory was set up within the suite of KCRI laboratories. In this space, serum, milk, vaginal or cloacal swabs, faeces, lymph nodes, kidneys, and meat can be safely processed without the fear of compromising other studies performed at this site. Establishment of this bespoke zoonoses laboratory at KCRI required considerable investment in time, funding, and relationship building as well as a combination of general infrastructure-focused or capacity-building grants and project-specific research grants; its future is dependent on continued income generation. A major benefit of establishing the zoonoses laboratory is that we can now collect and process both human and animal samples without the need to routinely ship material to other laboratories. This leads to more efficient generation of results; improved dissemination of result, for example, to participants, medical or veterinary stakeholders, researchers, and policymakers; enhanced response to findings; and a stronger sense of ownership in the source country. Further, the routine handling and analysis of samples builds and sustains skills and capacity within Tanzanian institutions, with potential for onward training and capacity strengthening in other institutions. For example, staff at the KCRI zoonoses laboratory now train technicians and researchers from other institutions, including government veterinary institutions, further enhancing cross-sectoral engagement and capacity.

**Photo 2.**
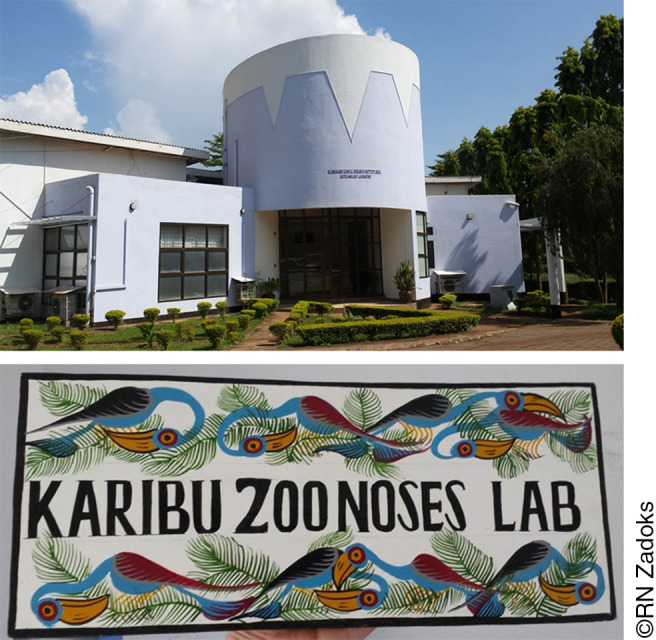
Kilimanjaro Clinical Research Institute, Moshi, Tanzania (top), and the welcome sign to the bespoke Zoonoses lab set up within KCRI to enable One Health research (bottom).

## PROGRESS IN ONE HEALTH PROJECTS

To demonstrate the value of establishing a One Health research platform, we describe some of our projects, which span the spectrum from wildlife and livestock to people and the values and perceptions that drive their behaviour.

### Zoonotic Causes of Febrile Illness

Febrile illness is a common cause of healthcare-seeking behaviour and is often attributed to malaria. Research conducted through the Kilimanjaro Christian Medical Centre-Duke University collaboration, however, showed that bloodstream infections and bacterial zoonoses caused more than half of all febrile illnesses in paediatric and adult patients.^6^ Bacterial zoonoses were 20 times more common than malaria as a cause of illness in febrile admissions, yet routine diagnostics for zoonoses are not available and many healthcare providers identify low knowledge and testing capacity as reasons for zoonoses under-diagnosis.^50^

Q fever and brucellosis, among other diseases, were commonly identified in febrile patients and may have a ruminant livestock reservoir. Within the genus *Coxiella*, the causative agent of Q fever, only a single species is known, *C. burnetii*, but different subtypes have been associated with cattle or small ruminants as primary sources for human infection.^51,52^ Within the genus *Brucella*, which causes brucellosis, multiple species are recognised: *Brucella abortus* is primarily found in cattle and *B. melitensis* in sheep and goats. This host–association is not absolute, and new *Brucella* species as well as the possibility of adaptation of known *Brucella* species to new host species at the wildlife–livestock interface is increasingly recognised.^53^ Thus, a range of questions arises: Which animal species contribute most to human infection and disease? Which pathways of transmission are most important? What are the critical points that can be targeted for interventions? How are health interventions and treatments perceived, understood and valued by livestock keepers? How do or could livestock handling practices, milk and meat supply chains, household preparation, and consumption practices, and livestock vaccination influence disease occurrence?

Serological tests cannot differentiate between infections caused by *B. abortus, B. melitensis*, or other *Brucella* species. Because different *Brucella* species have distinct transmission dynamics and control options, it is important to determine the identity of *Brucella* in patients with fevers and in potential reservoir host species. Achieving this requires access to the actual bacteria, which is difficult to obtain due to low levels of bacteremia in patients, transient bacterial shedding by infected animals, and constraints on culture of the organism. Using serological data from field studies and sophisticated modelling approaches, goats were implicated as the most likely source of human infection in northern Tanzania.^54^ This study also used simulated data to show that even small amounts of data on bacterial species identity—or comparable subtyping—are sufficient to inform models aimed at quantifying transmission between host species, thus justifying considerable investment in obtaining such data.^54^ Such efforts are now in progress, using human and animal sampling collection platforms set up in collaboration with human and veterinary medical professionals, and working with molecular bacteriologists in the zoonosis laboratory to prize this valuable information out of drops of urine, blood, and milk.

**Photo 3.**
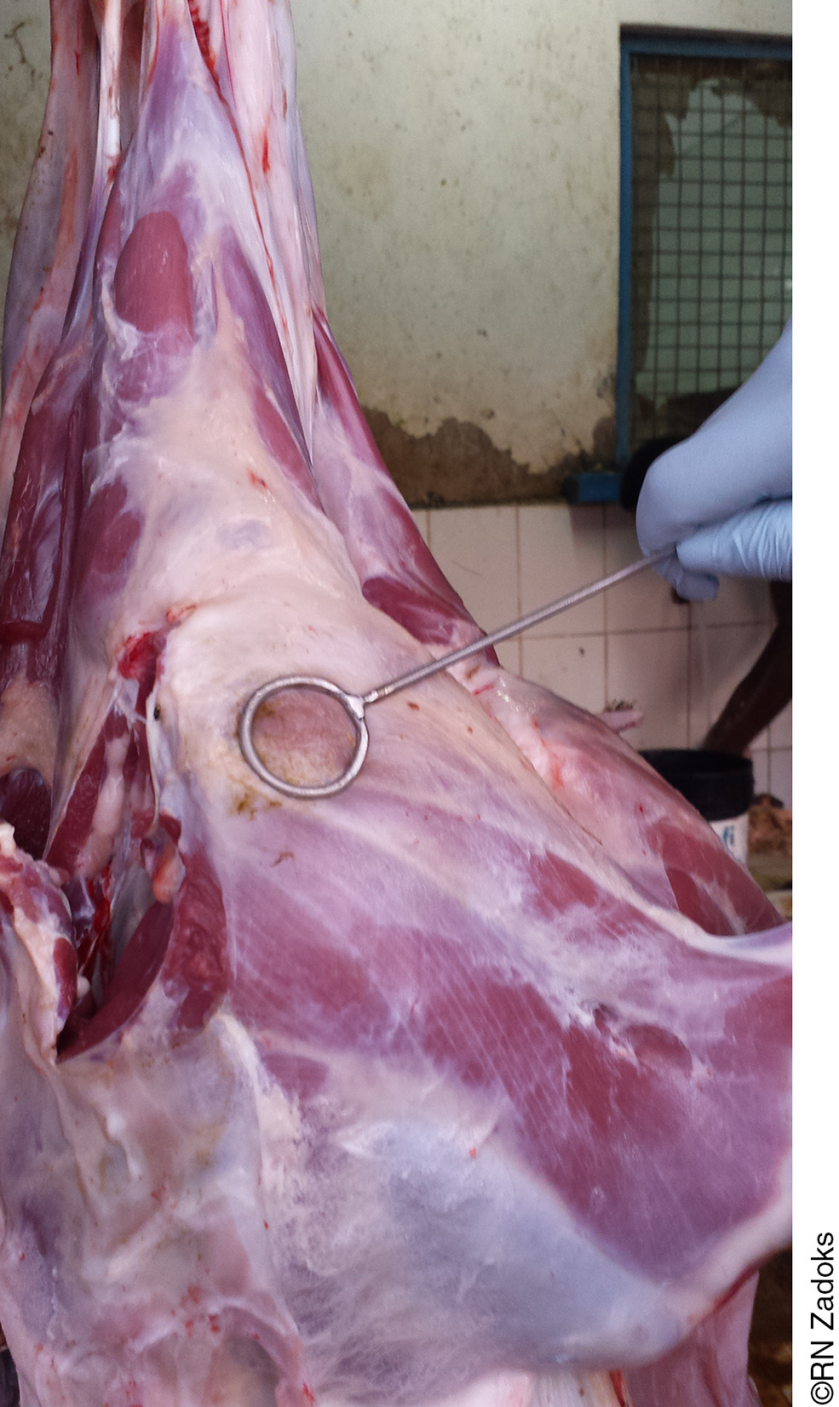
Staff training in standardized methodology for carcass swabbing to support slaughterhouse-based surveillance of meat borne pathogens such as non-typhoidal *Salmonella* and *Campylobacter* species. Moshi, Tanzania.

### Foodborne Zoonoses

Zoonoses can be transmitted through the milk supply and value chain as well as the meat supply and value chain. Live animals and animals at slaughter carry numerous bacteria that are harmless, or commensal, when they reside in the animal gut but are potentially dangerous pathogens once they find their way into people. This is particularly true for non-typhoidal *Salmonella* (NTS) and *Campylobacter*, which are—among other signs and symptoms—associated with invasive and often fatal disease and growth shortfalls, respectively.^55,56^ In Tanzania, *Salmonella* has been detected on carcasses of pigs and in fresh goat meat,^57,58^ while *Campylobacter* has been found in faeces or on carcasses of pigs, cattle, and ducks.^59,60^ People may acquire zoonotic *Campylobacter* from beef, particularly when meat preparation and processing is not undertaken properly,^61^ but it remains to be determined whether animals or food of animal origin contribute to the burden of human invasive NTS disease.^56,62^ Meat for consumption may become contaminated with faecal bacteria from the slaughtered animal, or with human- or animal-derived bacteria found in the environment of slaughter locations, butcheries, and eateries, such as on chopping blocks, butcher knives, or “nyama choma” (ready-to-eat meat). Targeted interventions require an understanding of the relative contribution of those potential sources of contamination and infection. Through a combination of supply- and value-chain analysis, mathematical modelling, and molecular epidemiology studies, we aim to identify the contributions of these different sources to human infections. The detection of NTS and *Campylobacter* in slaughter animals, carcasses, meat, and the environment is a crucial component of this work and is conducted in the zoonoses laboratory at KCRI.

### Anthrax

Anthrax is not normally thought of as a foodborne zoonosis, however, many outbreaks in sub-Saharan Africa and elsewhere have been attributed to meat consumption. In 2016, ProMed—a global electronic reporting system for outbreaks of emerging infectious diseases and toxins—reported cases or government warnings related to consumption of cattle in,^63^ for example, Kenya, Niger, Nigeria, Tanzania, and Zimbabwe. An outbreak in people in Zambia was linked to wildlife through the consumption of hippopotamus meat.^64^ While many Western scientists, regulatory agencies, and funding bodies think of anthrax as a biowarfare agent and impose very strict controls on anthrax research, it is an endemic disease in much of sub-Saharan Africa where it affects wildlife, livestock, and people.^65^ The spores of the causative bacterium, *Bacillus anthracis*, survive in the natural environment. Moreover, environmental factors drive interactions between wildlife, livestock, and humans, and between hosts and the environment, all of which impact the risk of disease.^66,67^ Communities generally know the risk of eating carcasses from animals that died from anthrax, and some have developed traditional methods to determine whether or not a carcass is safe to eat. When faced with a choice between a high likelihood of malnutrition and a small chance of falling sick, consumption of carcass meat is often considered a risk worth taking, sometimes resulting in fatal outcomes. Similarly, the risk of exposure to MCF from wildebeest may drive herders to take their cattle to alternative grazing areas despite a known risk of anthrax (O. R. Aminu, oral communication, November 2016). To gain a better understanding of the relationships between anthrax in wild-life, livestock, and people, we are working with communities in Ngorongoro Conservation Area (NCA) to map their knowledge of anthrax, its spatial distribution, and its direct and indirect impact on livestock and people. To make possible confirmation of anthrax diagnoses, we provide training to animal health workers in the NCA in safe sample collection methods and the use of personal protective equipment. Samples are shipped to KCRI for culture-free confirmation of the presence of anthrax and for genetic typing. Use of molecular tools will also enable us to identify newly emerging *Bacillus* strains such as *B. cereus* Biovar *anthracis*, which has been associated with anthrax-like disease in wildlife and livestock in numerous countries in West and Central Africa.^68,69^

**Photo 4.**
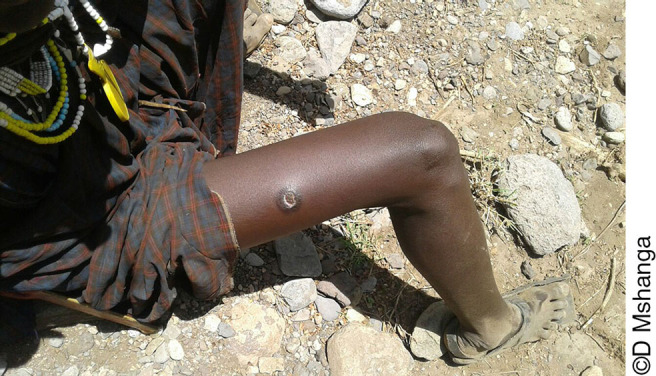
Cutaneous anthrax in a resident of the Ngorongoro Conservation Area where anthrax is an endemic disease with seasonal peaks in wildlife, livestock, and human case numbers.

## OUTLOOK

While the importance of One Health research is becoming increasingly recognised, it takes time and effort to break down the barriers between disciplines and their ways of working, and to build the interdisciplinary research teams, culture, and infrastructure needed to address One Health questions. Open Access publishing should reduce financial barriers to access to publications from other “silos”, but barriers in language and methodology will take longer to come down. In addition, institutional attitudes to ethical review may continue to be dominated by Western legislation and priorities, regardless of the country or culture where the research is conducted. Our experience in Tanzania suggests that when those barriers are overcome, the opportunities for One Health research open up. The importance of One Health research, and the potential for African countries to play a leading role in this arena, is recognised and encouraged by policymakers and by regional and international funding bodies alike. The establishment of the *East African Health Research Journal* provides an opportunity to disseminate and advocate for One Health research and is testament to the confidence this region has in its capabilities. To date, much of One Health research—particularlyinthesocialsciences—hasbeen led or carried out by non-African scientists. A range of initiatives aim to address this imbalance. Examples at the time of writing include the Zoonoses and Emerging Livestock Systems–Associated Studentship (ZELS-AS) program funded by Research Councils UK and the Department for International Development, with PhD studentships and supervision shared between high-, low-, and middle-income countries; the Leverhulme–Royal Society Africa Awards, which fund a PhD studentship and postgraduate training courses for East African scientists; the Program for Enhancing the Health and Productivity of Livestock (PEHPL), funded by the Bill and Melinda Gates Foundation, which supports non-western and western PhD students at the Nelson Mandela African Institution of Science and Technology (NM-AIST) in Arusha, Tanzania; and the Developing Excellence in Leadership, Training and Science (DELTAS) Africa program, which supports the Africa-led development of world-class researchers and scientific leaders in Africa. We hope that current and new generations of scientists will consider the *East African Health Research Journal* as a platform for their publications.
